# Case report: Primary chronic calcaneal bursitis treated with subtotal bursectomy in a cat

**DOI:** 10.3389/fvets.2022.915741

**Published:** 2022-09-12

**Authors:** YoungJin Jeon, Haebeom Lee, Yoonho Roh, Daehyun Kim, Seong Mok Jeong, Jaemin Jeong

**Affiliations:** ^1^College of Veterinary Medicine, Chungnam National University, Daejeon, South Korea; ^2^Division of Animal Surgery, Department of Clinical Veterinary Medicine, Vetsuisse Faculty, University of Bern, Bern, Switzerland

**Keywords:** calcaneal bursitis, subtotal calcaneal bursectomy, recurrent lameness, cyst-like mass, cat

## Abstract

A 6-year-old, female spayed Bengal cat with a bodyweight of 6.4 kg was presented with swelling of the bilateral calcaneal region and weight-bearing hindlimb lameness with a 4-month history of unsuccessful conservative therapy. On orthopedic examination, a cyst-like mass around the calcaneal tendon was palpated. Palpating the mass and flexing the tarsal joint triggered pain. Through ultrasonography and magnetic resonance imaging, an inflamed or fluid-accumulated lesion was suspected around the calcaneal tendon, but there was no evidence of calcaneal tendonitis. Swollen calcaneal bursae were removed surgically. Histopathologic examination revealed fibrosis and an edematous feature. The cat was diagnosed with bilateral chronic primary calcaneal bursitis based on history, clinical signs, and diagnostic results. Hence, subtotal bursectomy was performed. At 4 weeks postoperatively, the cat had no pain around the tarsal joints and was ambulating normally. Radiographic and ultrasonographic exams revealed no recurrence of swelling or inflammation in the calcaneal region. Thirteen-month follow-up confirmed acceptable function and no relapse of clinical signs. The inflammation of calcaneal bursa alone can be the primary cause of hindlimb lameness in cats. A cat with hindlimb lameness and swelling on the calcaneal region should be assessed with the possibility of primary calcaneal bursitis. Subtotal calcaneal bursectomy can be considered as an effective treatment for primary chronic bursitis.

## Introduction

Synovial bursa is a small, flattened sac of synovial membrane located between the tendons, muscle, or skin at points of friction or stress. Bursa helps to reduce friction and provides freedom of movement over a short distance (1). Bursitis is an inflammatory condition of the bursa. Calcaneal bursitis has been described mainly in humans and large animals such as equine and bovine (2–5). Clinical signs of the condition include lameness of the affected limb and swelling over the bursa.

In small animals, bursitis has rarely been reported. One case of chronic olecranon bursitis in a dog has been described. Conservative therapy such as aspiration and intralesional administration of dexamethasone and hyaluronidase were tried, but there was no effect. The dog was treated with resection of the bursa, and the outcome was successful (6). There have been reports of luxation of superficial digital flexor tendon causing calcaneal bursitis in a dog and a cat (7, 8). The cat was treated surgically with the removal of the inflamed calcaneal bursae, and the outcome was successful (8).

However, primary calcaneal bursitis has not been reported in small animals. Therefore, we present the first case of bilateral chronic primary calcaneal bursitis eliciting hindlimb lameness in a cat. This case report describes the successful surgical management of primary calcaneal bursitis with subtotal bursectomy in a cat.

## Case description

**Figure 1:**
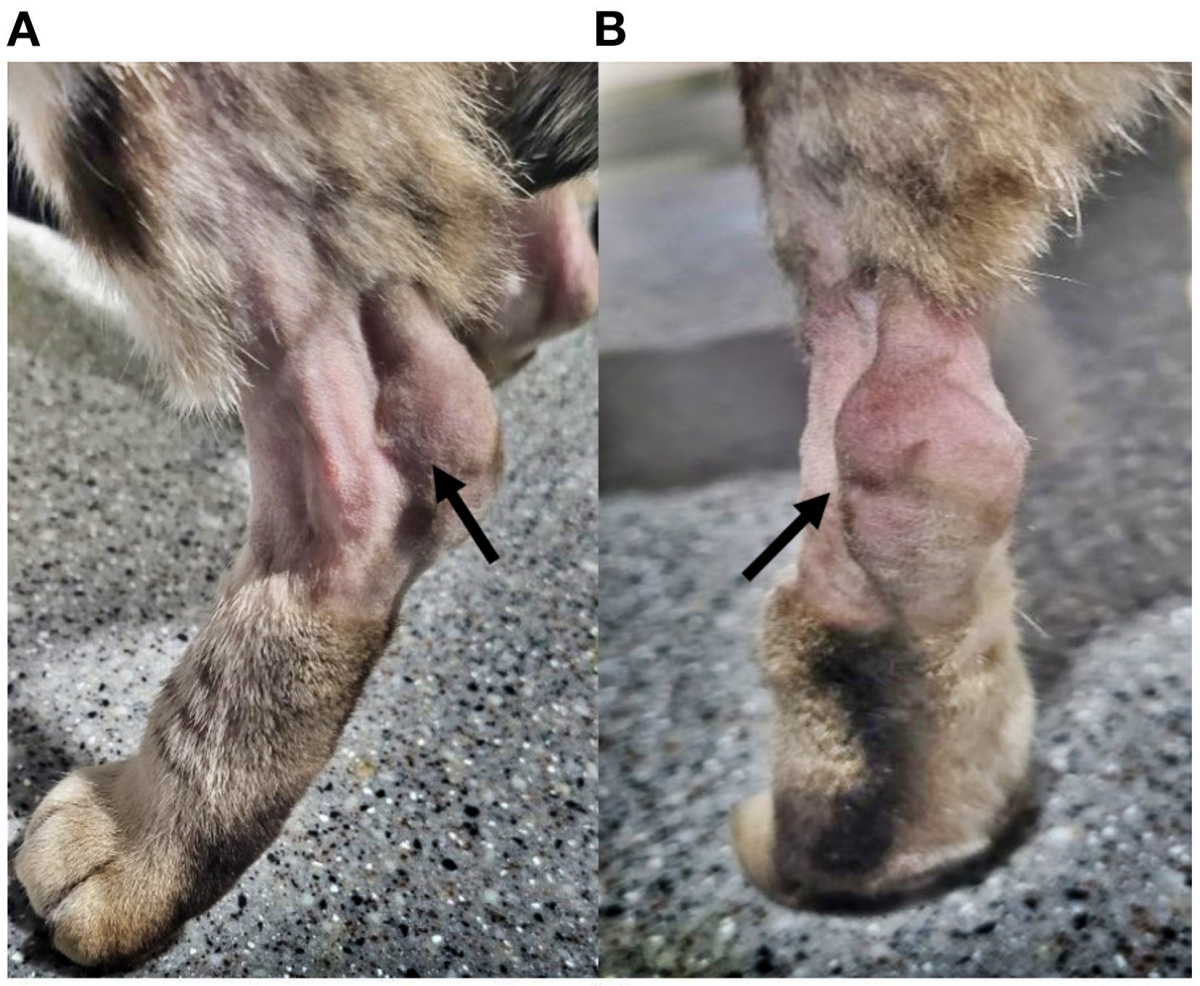
Gross appearance. Gross appearance of the calcaneal region at the initial evaluation. Hair around the region has been clipped for assessment. **(A)** Lateral view of the left calcaneal region (arrow). **(B)** Caudocranial view of the left calcaneal region. Remarkable swelling is confirmed around the calcaneal tuberosity (arrow).

A 6-year-old, female spayed Bengal cat weighing 6.4 kg was presented for evaluation of the swollen left calcaneal region and weight-bearing hindlimb lameness. The patient showed left hindlimb limping and had difficulty jumping or going up stairs. There was no history of trauma. A cyst-like mass and swelling around the calcaneal tendon were revealed on physical examination (Figure 1). Manipulating the mass and flexing the tarsal joint revealed tenderness. The calcaneal tendon and its component tendons were not luxated.

**Figure 2:**
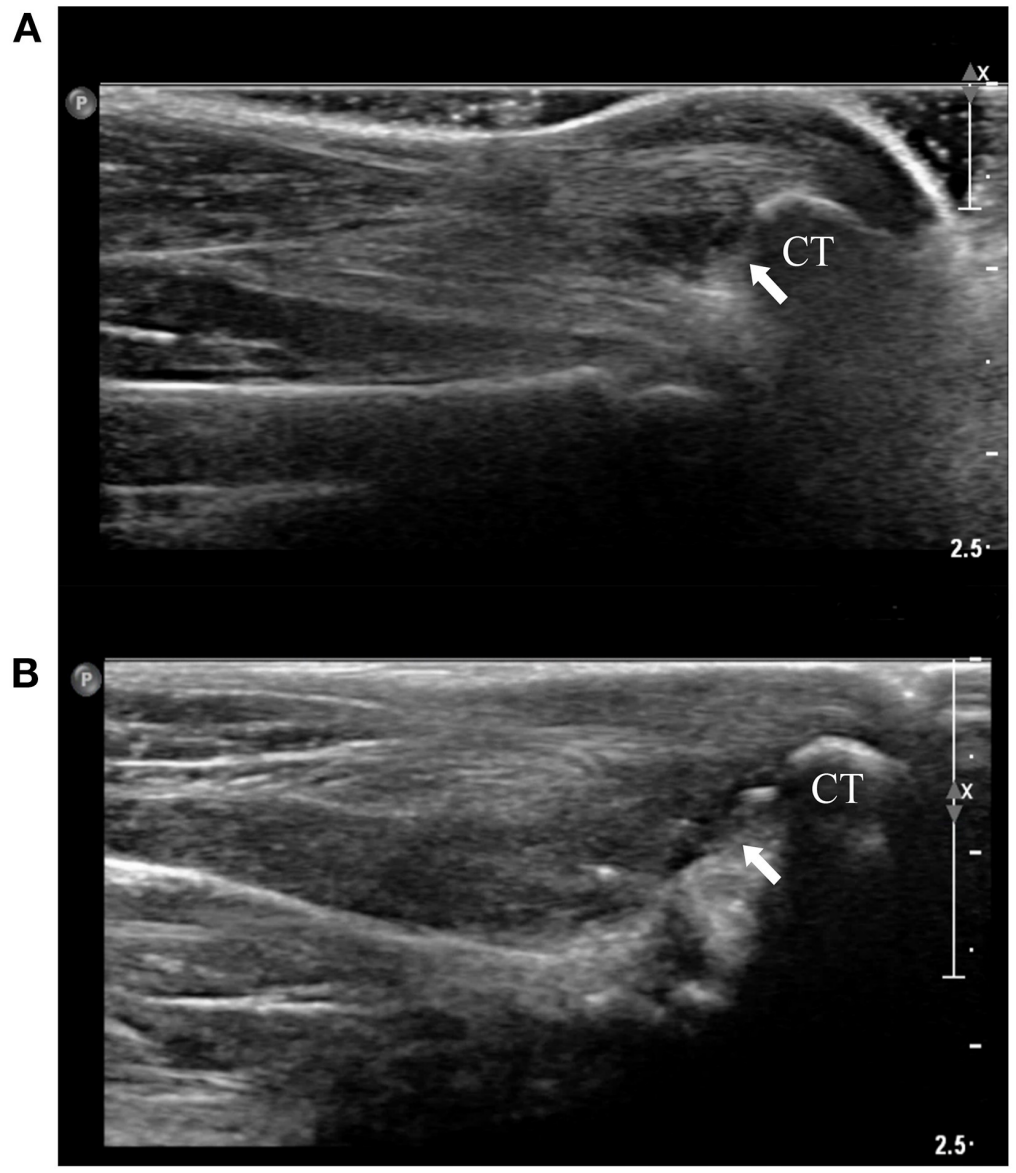
Ultrasonography. **(A)** Longitudinal view of left calcaneal tendon and calcaneus at the initial evaluation. There is a heterogeneous hypoechoic lesion (arrow), indicating moderate fluid accumulation and/or inflammation within the bursa. **(B)** Longitudinal view of left calcaneal tendon and calcaneus at 4 weeks postoperatively. Absence of the lesion can be confirmed (arrow). Calcaneus tuberosity was indicated as CT on the figures.

Mediolateral and craniocaudal radiographic images revealed increased opacity between the distal tibia and calcaneal tendon. On ultrasonography (Figure 2A), hypoechoic fluid-filled lesions were located at the normal anatomic position of the gastrocnemius calcaneal bursa, but evidence of calcaneal tendonitis could not be identified.

Conservative therapy was initiated; the fluid was aspirated and followed by meloxicam oral suspension (0.05 mg/kg orally once daily) and prophylaxis antibiotics, including amoxicillin-clavulanate (12.5 mg/kg orally twice daily) and clindamycin (10 mg/kg orally twice daily) for 7 days. Microscopic examination of the aspirated fluid revealed low nucleated cell density (<3 cells/hpf) and most of them were mononuclear cells (90%) with a small number of neutrophils (10%). There was no sufficient amount of fluid to perform quantitative cell counts. The fluid was incubated aerobically and anaerobically for 3 days, and no organisms (fungus included) were isolated. The patient was able to ambulate without limping, and jump upstairs after the aspiration. Meloxicam was replaced by PDS (2 mg/kg orally twice daily for 2 months) after the washout period of 7 days. Tramadol (2 mg/kg orally twice daily) was added as an analgesic, and both the medications were tapered for 1 month. Despite the conservative treatment, 1 month after the first visit, the same clinical conditions occurred contralaterally (right calcaneal region). Aspiration of the fluid was repeated, and the patient showed improvement of the clinical signs. However, recurrence of swelling of the bilateral calcaneal bursa occurred 2 months after the last aspiration.

**Figure 3:**
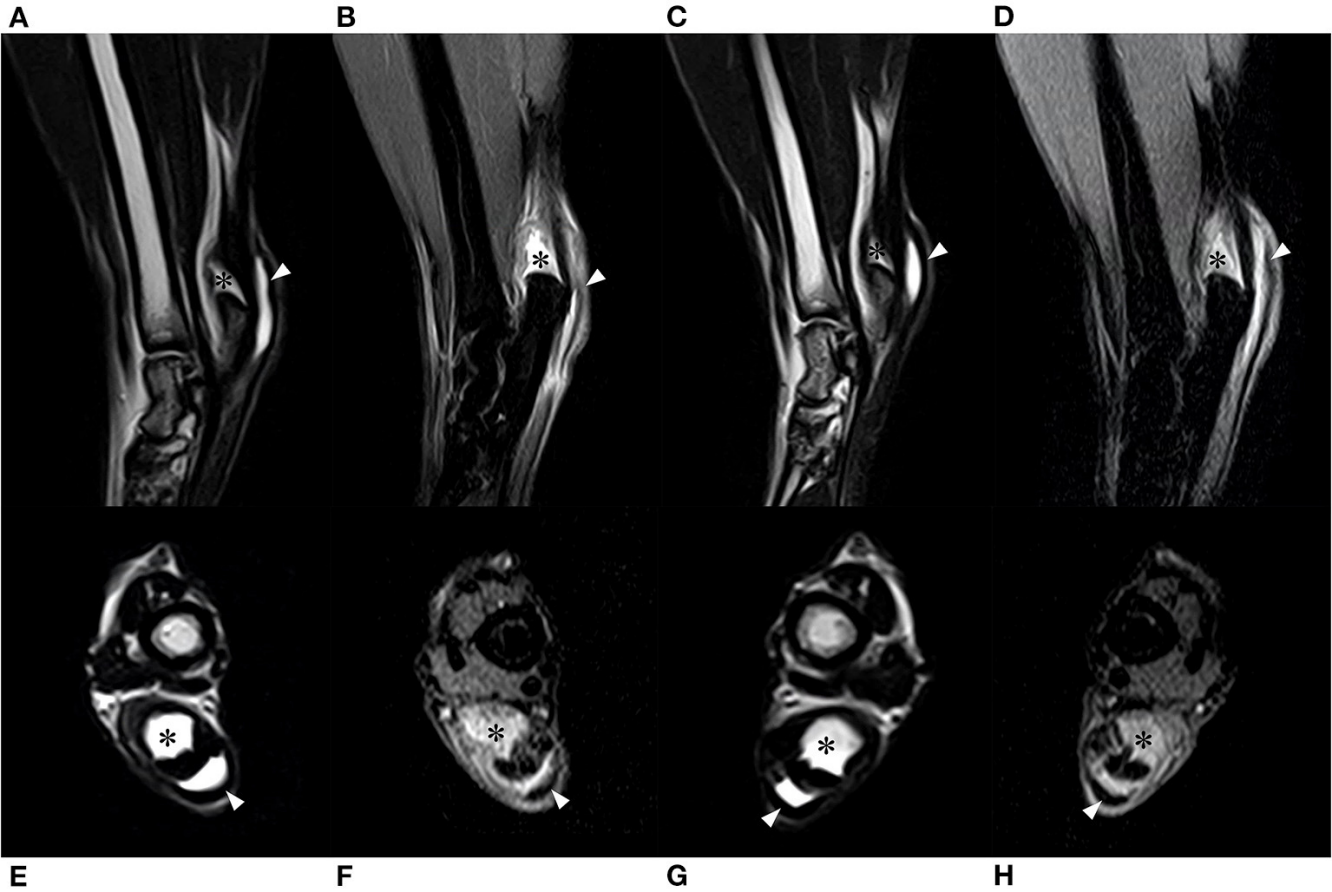
Magnetic resonance imaging. **(A)** T2-weighted sagittal image of the right tarsal joint. **(B)** Postcontrast fat saturated proton density-weighted sagittal image of the right tarsal joint. **(C)** T2-weighted sagittal image of the left tarsal joint. **(D)** Postcontrast fat saturated proton density-weighted sagittal image of the left tarsal joint. **(E)** T2-weighted coronal image of the right tarsal joint. **(F)** Postcontrast fat saturated proton density-weighted coronal image of the right tarsal joint. **(G)** T2-weighted coronal image of the left tarsal joint. **(H)** Postcontrast fat saturated proton density-weighted coronal image of the left tarsal joint. The gastrocnemius calcaneal bursa (asterisk) and intertendinous bursa (arrowhead) were identified as hyperintense regions. Components of the calcaneal tendon such as common tendon, gastrocnemius tendon, and superficial digital flexor tendon were intact (identified as hypointense string).

For further investigation, magnetic resonance imaging (MRI) was performed (Figures 3A–H). Inflammation and accumulation of fluid were suspected in the bilateral gastrocnemius calcaneal bursa and intertendinous calcaneal bursae. The tendons and muscles around the bursae were intact.

**Figure 4:**
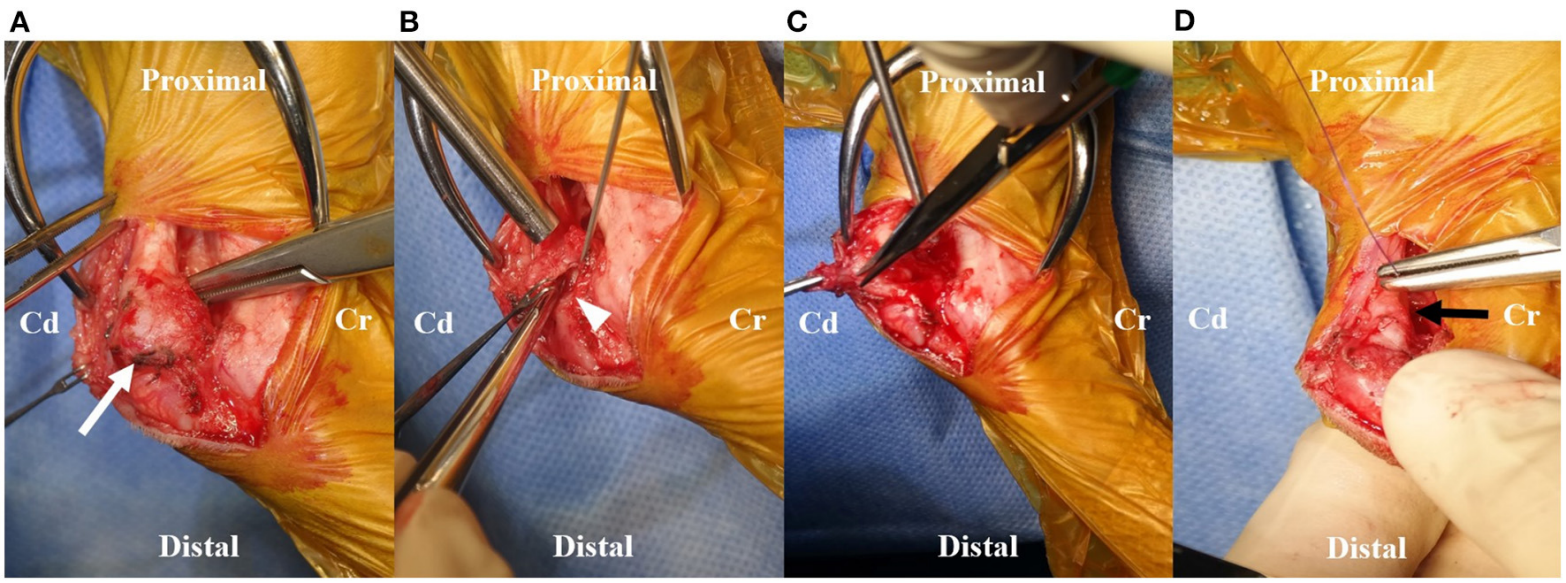
Intraoperative images. **(A)** Prominently enlarged gastrocnemius calcaneal bursa (white arrow) was identified intraoperatively. **(B)** The serous fluid was released from the incised opening (white arrowhead) on the bursa using a No.11 blade. The opening of the bursa was extended by Castroviejo scissors. **(C)** The dissection using tenotomy scissors was performed until the normal Achilles tendon was isolated from the bursa. The redundant bursa was removed. **(D)** The medial retinaculum was sutured to the edge of the tendon using monofilament, absorbable suture material. Note the appearance of normal Achilles tendon after removal of gastrocnemius calcaneal bursa with shrinkage of the swollen lesion (black arrow). Cd, Caudal; Cr, Cranial.

A tentative diagnosis of chronic calcaneal bursitis was made. Bilateral subtotal bursectomies were planned for removal of inflamed chronic lesions including gastrocnemius and intertendinious calcaneal bursae. After following general anesthesia, a caudomedial incision was made through the skin over the right calcaneus for approximately 4 cm. The medial retinaculum was dissected for approaching the lesions. The calcaneal tendon was surrounded by distended bursa tissue, and the gastrocnemius calcaneal bursa was especially enlarged (Figure 4A). The bursa was dissected from the surrounding tissue and incised carefully using a no. 11 blade, and the serous fluid was released (Figure 4B). The incision was extended using Castroviejo scissors and small tenotomy scissors. The redundant bursa was separated and removed by blunt and sharp dissection until normal Achilles tendon was confirmed (Figure 4C). The medial retinaculum was apposed to the edge of the tendon with monofilament, absorbable suture material (PDS plus USP 3-0, Ethicon, Raritan, NJ, USA) using a horizontal mattress suture (Figure 4D). Skin closure was performed routinely. The same procedures were performed on the contralateral side. Manipulation of the tarsal joint was performed postoperatively, and decreased range of motion or impingement of the calcaneal tendon was not observed. Two-layer bandage was used to protect both the wounds, which were wrapped with adhesive tape.

Postoperative medical management was initiated with meloxicam suspension (0.05 mg/kg orally twice daily), tramadol (2 mg/kg IV twice daily), and cefazolin (22 mg/kg IV twice daily). The patient recovered uneventfully and was discharged 9 days postoperatively with the medications. Tramadol (2 mg/kg orally twice daily) and gabapentin (10 mg/kg orally twice daily) were prescribed for 2 weeks and tapered for 2 weeks as pain management medication. Silymarin (7.5 mg/kg orally twice daily) was added as hepatic supplement. The patient's owner was educated regarding postoperative care including restricted activity for 4 weeks and rehabilitation once a week for 8 weeks.

**Figure 5:**
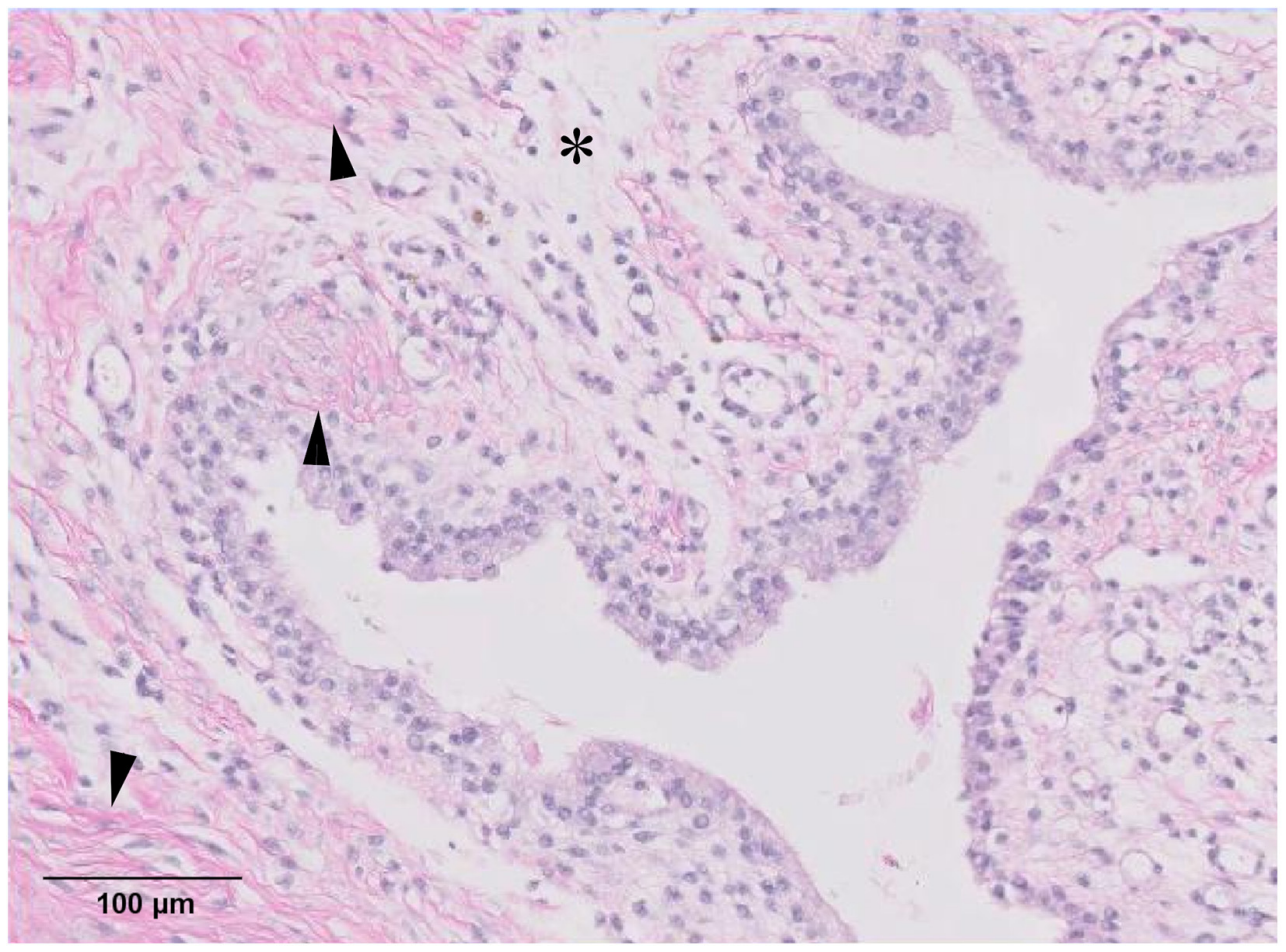
Biopsy results. Synovium with adjacent fibrous connective tissue, adipose tissue, and vasculature. Moderate fibrosis (arrowhead) is noted, and moderately edematous lesions (asterisk) are identified (magnification ×20, haematoxylin and eosin stain).

Histopathologic examination revealed that the specimens consisted of synovium with connective tissue with mild to moderate fibrosis (Figure 5). There were no evidence of infection or neoplasia. The swelling of the calcaneal region and lameness were resolved 4 weeks postoperatively. Manipulation of the tarsal joint did not reveal tenderness. Radiographic and ultrasonographic evaluation of calcaneal region revealed reduced opacity between distal tibia and calcaneal tendon and absence of heterogenous hypoechoic lesions cranial to calcaneal tuberosity (Figure 2B). The patient did not reveal discomfort when the surgical site was palpated 3 months postoperatively. Telephonic follow-up with the patient's guardian at 13 months after surgery confirmed that the cat was ambulating without any signs of lameness or evidence of relapse.

## Discussion

This report described diagnosis and surgical treatment of bilateral primary calcaneal bursitis in a cat. We ruled out the known causes of calcaneal bursitis, including trauma, superficial digital flexor tendon luxation, and calcaneal tendonitis through an orthopedic exam and diagnostic imaging, including radiography, ultrasonography, and MRI (6–9). Furthermore, the other causes like infection or neoplasm were ruled out by cytology, and histopathologic examination (9–12). Although we could not identify the cause of the disease, to our knowledge, this is the first report on primary calcaneal bursitis. A bilateral subtotal bursectomies were performed to treat primary calcaneal bursitis. The clinical outcome showed excellent hindlimb function until 13 months postoperatively.

In human medicine, inflammatory disease of the bursa tendinis calcanea is termed retrocalcaneal bursitis. An abnormal enlargement of the bone where the Achilles tendon is inserted is called Haglund's deformity, which is known to be a predisposing factor for retrocalcaneal bursitis (5). Haglund's deformity can induce high pressure in the retrocalcaneal bursa, which is suspected to be the main pathological factor (4). There is no report of bony deformation of calcaneal process causing pain of the tarsal joint in cats. In this case, no evidence of abnormal bony structure of calcaneus was found when compared with the mediolateral tarsal joint view X-ray of normally ambulating cats.

The treatment of retrocalcaneal bursitis starts with the conservative method. It includes oral anti-inflammatory medications, antibiotics, aspiration, topical lidocaine, local steroid injection and physiotherapy such as exercise and electric shock wave therapy (5, 13, 14). In a human study, 15–50% of conservatively treated patients did not respond well and proceeded to surgery (4). Although conservative treatment should be tried first in all cases, surgical treatment should be considered within 1 year of onset of the disease (15). Surgical treatment of retrocalcaneal bursitis focuses on decreasing compression of the retrocalcaneal bursa or/and removal of the bursa. Approximately 73–91% of patients who underwent surgical treatment had either excellent or good satisfaction (3).

In large animals, traumatic events with or without infection have been reported as a common cause of calcaneal bursitis (9–11). Calcaneal bursitis not responding to conservative therapy was suggested to be treated with surgical management, including debridement, synovial lavage, and bursectomy in veterinary medicine (6, 8–11). Initially, our case was treated by conservative therapy, and clinical signs were resolved. However, recurrence of the clinical signs occurred, and bilateral bursectomies were performed. Although the bursa was known to reduce friction between the tendons, muscles, or skin, the patient showed no tenderness around the tarsal joint and no evidence of Achilles tendonitis after the bursectomy (1). The patient could ambulate almost normally without recurrence of the bursitis until the 13 months of follow-up.

This case report has some limitations. First, we reported only a single case. Although an excellent clinical outcome was obtained from our case without any complications, an extended long-term follow-up with large case series is needed to evaluate the benefits of subtotal bursectomy (16). Secondly, we could not elucidate the pathomechanisms of bilateral chronic primary calcaneal bursitis. Finally, further studies are necessitated to evaluate the biomechanical effect of a subtotal calcaneal bursectomy on the tarsal joint movement.

## Conclusion

This case report describes for the first time the clinical features, diagnosis and therapeutic management of a chronic primary calcaneal bursitis in a cat. We propose that the subtotal bursectomy could be an effective treatment in cases unresponsive to conservative treatment. Further clinical studies are necessary to investigate the etiology and clinical outcome of calcaneal bursitis treated with subtotal bursectomy.

## Data availability statement

The raw data supporting the conclusions of this article will be made available by the authors, without undue reservation.

## Ethics statement

Ethical review and approval was not required for the animal study because this study is a case report of examination, surgery, and biopsy performed for the purpose of treatment of the patient, and no action contrary to treatment was performed. Written informed consent was obtained from the owners for the participation of their animals in this study.

## Author contributions

YJ, JJ, and HL actively involved in managing the case and writing and editing the manuscript. HL performed the surgery. JJ reviewed the article critically and revised the manuscript. YR, DK, and SJ supervised the clinical management of the case. All authors contributed to preparation and final approval of the manuscript.

## Conflict of interest

The authors declare that the research was conducted in the absence of any commercial or financial relationships that could be construed as a potential conflict of interest.

## Publisher's note

All claims expressed in this article are solely those of the authors and do not necessarily represent those of their affiliated organizations, or those of the publisher, the editors and the reviewers. Any product that may be evaluated in this article, or claim that may be made by its manufacturer, is not guaranteed or endorsed by the publisher.

## Abbreviations

MRI: magnetic resonance imaging
Cd: caudal
Cr: cranial.
